# Quantification of *N*-phenyl-2-naphthylamine by gas chromatography and isotope-dilution mass spectrometry and its percutaneous absorption ex vivo under workplace conditions

**DOI:** 10.1007/s00204-017-2046-2

**Published:** 2017-09-12

**Authors:** Eike Maximilian Marek, Stephan Koslitz, Tobias Weiss, Manigé Fartasch, Gerhard Schlüter, Heiko Udo Käfferlein, Thomas Brüning

**Affiliations:** Institute for Prevention and Occupational Medicine of the German Social Accident Insurance, Ruhr-University Bochum (IPA), Bürkle-de-la-Camp Platz 1, 44789, Bochum, Germany

**Keywords:** *N*-Phenyl-2-naphthylamine, *N*-Phenyl-β-naphthylamine, Franz diffusion cells, Percutaneous penetration, Skin

## Abstract

*N*-Phenyl-2-naphthylamine (P2NA) is an antioxidant used to protect rubbers from flex-cracking. P2NA can be converted in vivo to 2NA, one of the most potent bladder carcinogens. Here, we report the specific and ultra-sensitive quantification of P2NA in the receptor fluid of Franz diffusion cells by gas chromatography and isotope-dilution tandem-mass spectroscopy (GC–MS/MS). The experimental conditions were optimized to minimize losses of P2NA due to surface absorption on glass, plastic, and rubber material, and subsequently validated. Static and dynamic diffusion cell conditions were used to study the percutaneous penetration of P2NA into freshly prepared porcine skin. The experimental settings closely resembled those of the printing industry in the 1960s/1970s in Germany where P2NA-containing solutions in dichloromethane have been used. P2NA penetrated the skin at very low levels (0.02 ± 0.01 µg/cm^2^/h) with a cumulative penetrated amount of 0.80 ± 0.26 µg/cm^2^, a lag time of 6.33 ± 2.21 h and under dynamic conditions. Compared to the receptor fluid, 10–40-fold higher concentrations were found in the skin, predominantly in the dermis and the stratum corneum. Dichloromethane acted as a penetration enhancer by increasing the cumulative penetrated amounts and the recovery of P2NA in both the receptor fluid and the skin, while shortening its lag time. However, the flux remained unaffected. Due to its accumulation in subcutaneous layers, we finally proved that P2NA is continuously released into the receptor fluid despite exposure cessation up to 160 h. Overall, the results show that close attention has to be paid to dermal absorption of P2NA in exposed workers.

**Electronic supplementary material:**

The online version of this article (doi:10.1007/s00204-017-2046-2) contains supplementary material, which is available to authorized users.

## Introduction

*N*-Phenyl-2-naphthylamine (P2NA) is a lipophilic secondary aromatic amine and has been used in industry as an antioxidant until the 1980s to protect natural and synthetic rubbers and latexes from thermal degradation, oxidation, and flex-cracking (IARC 1982). Furthermore, P2NA was used as antioxidant in greases, lubricating oils, and transformer oils (Tolbert 1997).

P2NA was categorized in 1987 as a group 3 carcinogen (not classifiable as to its carcinogenicity to humans) by the International Agency for Research on Cancer (IARC) due to “*inadequate evidence for carcinogenicity to humans*” and “*limited evidence for carcinogenicity to animals*” (IARC 1987). Recent epidemiological studies still remain inconclusive regarding the carcinogenic risk of P2NA in humans (Sorahan 2008; Sorahan et al. 2000). However, dephenylation of P2NA to 2-naphthylamine (2NA), one of the most potent bladder carcinogens (IARC 2012), has been reported in rats and dogs (Weiss et al. 2013; Laham and Potvin 1983; Batten and Hathway 1977), and based on the detected amounts of 2NA in urine after oral dosage of P2NA, is also assumed in humans (Kummer and Tordoir 1975; Moore et al. 1977). Due to the metabolic conversion of P2NA to 2NA, the high carcinogenic potency of 2NA, the long latency period of urothelial cancer, and the assumed on-going use of P2NA as an antioxidant in natural and synthetic rubber industry outside western industrial nations, P2NA-associated bladder cancer at the workplace is still of relevance to the present day (Weiss et al. 2007).

The supposed main exposure routes to P2NA in industry are by inhalation (e.g., when handling powdery products) or by dermal contact (e.g., when using P2NA-containing solvents or oils) (IARC 1982). Previously, it has been reported that P2NA was unable to penetrate human skin when using Franz diffusion cells (Wellner et al. 2008), an accepted model to study the dermal absorption of potentially harmful chemicals in vitro (Bartosova and Bajgar 2012).

Here, we report the specific and ultra-sensitive quantification of P2NA in the receptor fluid of Franz diffusion cells by gas chromatography and isotope-dilution tandem-mass spectroscopy (GC–MS/MS) after percutaneous penetration through freshly prepared porcine skin. For this purpose, deuterated P2NA (P2NA-d_5_) was synthesized and used as an internal standard (ISTD). In addition, various materials used in Franz cell experiments and the composition of different receptor fluids were tested to minimize losses of P2NA due to surface absorption. A pre-validation in terms of comparing the porcine skin model with previously published data in human skin and using the primary aromatic amines aniline and *o*-toluidine showed that the results obtained by both models are fully comparable, confirming the current OECD guidelines. When applying a typical exposure scenario in the printing industry from the late 1970s where P2NA-containing solutions of dichloromethane (DCM) were applied by hand, we ultimately show that toxicologically relevant amounts of P2NA can penetrate the skin. We also report that P2NA accumulates in the subcutaneous layers of the skin. We finally show that, in spite of removing the P2NA-containing donor solutions from the skin surface, P2NA is continuously released out of this subcutaneous depot into the receptor fluid and, for this reason, can additionally contribute to the internal body burden of exposed persons.

## Materials and methods

### Chemicals

P2NA (CAS 135-88-6; purity >97%) was purchased at TCI GmbH (Eschborn, Germany). Ethanol (EtOH), dichloromethane (DCM), *n*-hexane, acetonitrile, toluene (all of analytical grade), potassium hydroxide (KOH), and trypsin were obtained from Merck KGaA (Darmstadt Germany). Deionized water was prepared by a Millipore Quantum Advantage system (Merck-Millipore, Darmstadt, Germany). The 0.9% solution of sodium chloride (NaCl) was obtained by Fresenius Kabi GmbH (Bad Homburg, Germany), whereas bovine serum albumin (BSA, fraction V lyophilized powder) was purchased by VWR International (Darmstadt, Germany). Stable isotope labelled P2NA-d_5_ was synthesized according to Bin et al. (2014) and provided by Dr. V. Belov (Max Planck Institute for Biophysical Chemistry).

### Franz diffusion cells

Diffusion cells according to Franz (Franz 1975) with six cells in parallel have been used (SES GmbH, Bechenheim, Germany). PTFE gasket rings (C. Otto Gehrckens GmbH, Pinneberg, Germany) were used to fix the skin samples on the donor side (3.14 cm^2^). A constant physiological skin temperature (32 °C) was ensured by heating the receptor chamber with a thermostatic circulation water bath (Thermo-Scientific, Dreieich, Germany) and using a magnetic stirrer. Depending on the tested study scenarios static and dynamic Franz cell conditions were used (see below).

### Preparation and use of skin samples

Fresh porcine skin samples (*sus crofa domestica*) were obtained prior to scalding from the inner (*n* = 3) and outer side (*n* = 3) of the ear for each ex vivo experiment (Meyer et al. 2007). The ears were washed (cold water) and clipped, and the skin was prepared with a recommended thickness of ~1 mm (OECD 2004) using a dermatome (Humeca, Enschede, The Netherlands). The reproducibility of the preparation and the physical integrity of the skin were tested by HE staining and bright-field microscopy. In addition, the barrier function of the porcine ear skin was compared to human skin of the inner and outer side of the hands by measuring the transepidermal water loss (TEWL, Antonov et al. 2016; Hui et al. 2012). After all percutaneous experiments, the skin samples were separated into three fractions [stratum corneum (*SC*), epidermis, and dermis] as previously described (Turksen 2005). For this, the skin was treated with a 2.5% tryptic solution (24 h, 36 °C). The *SC* and dermis were separated by forceps, whereas the epidermal cells remained in suspension. The dermis was saponified (5 mL 1 N KOH solution, 4 h). 20 µL ISTD were added to 500 µL of each skin extract and the samples were prepared and analysed as described below.

### Optimization of the ex vivo system

To minimize surface absorption of P2NA, three different materials [glass, polypropylene (PP), Tygon^®^-tubing (PTFE)], and four different receptor fluids [0.9% NaCl, 5% BSA, 0.9% NaCl +5% BSA, EtOH/water (50/50, v/v)] have been tested. In addition, the stability of P2NA in 5% BSA and EtOH/water (50/50, v/v) was tested up to 24 h at RT (21 °C) and 37 °C and in both glass and PP vessels. The potential degradation of P2NA during freezing/thawing-cycles has been studied up to 6 days. Finally, P2NA absorption has been studied for PTFE and fluororubber gasket rings, which are used to insert the skin samples in the Franz cells. For this purpose, each ring, which was treated with a P2NA-solution in a glass container (*n* = 6), was extracted by *n*-hexane and the samples were analysed as described below. All experiments were carried out using a solution of 100 µg P2NA/L and by triplicate analyses unless otherwise noted.

### Calibration standards and quality control

A stock solution of P2NA (1 g/L) was used to prepare two standard solutions (0.5 and 5 mg/L), all in acetonitrile. Then, seven calibration standards (2.5–250 µg/L) in EtOH/water (50/50, v/v) were prepared. Similarly, a stock solution of P2NA-d_5_ (1 g/L) was used to prepare an internal standard (ISTD) solution (2 mg/L) in acetonitrile. The calibration standards were processed as described in sample preparation. The calibration curves were plotted in terms of the ratio standard/internal standard (AUC) versus the concentration by the MassHunter Software (Agilent Technologies, Waldbronn, Germany). As no quality control material is commercially available, two P2NA solutions (10 and 30 µg/L) in 0.9% NaCl +5% BSA were prepared, similar to the calibration standards. Each analytical series contained a full set of calibration standards, one of each quality control sample and a reagent blank.

### Sample preparation

Samples from the percutaneous experiments (500 µL) were diluted with 4.5 mL of an EtOH/water mixture (50/50, v/v) to a final volume of 5 mL, whereas 5 mL of the calibration standards were used as such. Twenty microlitre of the ISTD was added to each sample. Liquid/liquid extraction of P2NA was carried using 6 mL of *n*-hexane by shaking (10 min) and centrifugation (10 min at 2100 g). The extract was transferred to a 20-mL screw-top vial and evaporated to ~1 mL in a rotational vacuum concentrator (RVC 2-33 CDplus, Martin Christ GmbH, Osterode, Germany). Finally, the sample was transferred to a 1-mL screw-top vial which contained 100 µL of toluene, vortex mixed, evaporated to approximately 100 µL by vacuum concentration, and transferred to a micro insert for analysis.

### GC–MS/MS analysis

Analysis was carried out by gas chromatography (GC, 7890A) and triple quadrupole mass spectrometry (MS/MS, 7000A). One microliter was injected splitless (injector temperature 300 °C). Separation was performed on a DB-5MS capillary column (phenyl-arylene polymer, 30 m, ID 0.25 mm, film 0.25 µm, J&W Scientific). Helium 6.0 was used as the carrier gas. The temperature program was 70 °C (3 min), 10 °C/min to 120 °C (1 min), 40 °C/min to 280 °C (0.5 min), and 40 °C/min to 320 °C (3 min). The transfer line, ion source, and quadrupole temperatures were set to 280, 230, and 150 °C, respectively. The flow rate of the collision gas (nitrogen 5.0) and the quench gas (Helium 6.0) were 1.5 and 2.25 mL/min. Quantification and confirmation were carried out by multiple reaction monitoring (EI-MRM, 70 eV) with *m/z* 219→217 (quantifier) and *m/z* 219→115 (qualifier) for P2NA, and *m/z* 224→224 (parent mass) and 224→221 for P2NA-d_5_.

### Analytical reliability

The limit of detection (LOD) and the limit of quantitation (LOQ) of the method have been determined as *S/N* ratio of three and nine. The intra- and inter-day imprecisions and the recovery were determined in a 5% BSA solution at 10 and 30 µg/L (*n* = 6 each).

### Test scenario 1

Workers in the printing industry were using up to 1% P2NA solutions in 96% DCM and 4% turpentine oil when refreshing rubber rolls (=protecting them from flex-cracking during use). The solutions were applied on the rubber rolls approximately four times per 8-h-shift and 5 min each. These tasks were usually carried out by hand and often without personal protection measures, such as the use of solvent-resistant gloves. Consequently, we used 0.5 mL of a 1% P2NA solution (12 g/L, 1.91 mg/cm^2^) in our initial experiments. In addition, the donor solution was applied for 1 h on the skin only. To prevent evaporation of DCM, the donor chamber was sealed with in-house produced glass chambers. After 1 h, the donor solution was removed, the skin in the donor cell cleaned (rinsed), and an equivalent amount of 5% BSA solution without P2NA was added. Then, the penetration of the initially applied P2NA was further assessed up to 48 h. The experiments were carried out using static (receptor chamber filled with 7.5 mL 5% BSA solution) and dynamic conditions (7.5 mL/h, 5% BSA solution) in freshly prepared porcine skin. Please note that the removal of the donor solution after 1 h is not consistent with OECD guidelines. However, this scenario most closely resembled the workplace situations in the printing industry in the 1960s/1970s.

### Test scenario 2

In line with OECD guidelines (OECD 2004), we repeated test scenario 1 without removal of the donor solution (application time = observation time = 48 h). Again, the experiments were carried out using static and dynamic conditions and freshly prepared porcine skin.

### Test scenario 3

To study the influence of DCM/corn oil on the percutaneous absorption of P2NA, we directly compared a 5 mg/L donor solution of P2NA in 0.9% NaCl/5% EtOH with a solution in DCM/corn oil (96/4). All other experimental settings were kept constant, i.e., dynamic conditions, freshly prepared porcine skin samples, and an application time of 48 h were used.

### Skin penetration parameters

The cumulative penetrated amount (CPA) of P2NA was calculated according to

$$m_{{{\text{P2NA}} t_{n} }} \left[ {{{\upmu \text{g}}}} \right] = \frac{{c_{{{\text{P2NA}} t_{n} }} \,[{{\upmu \text{g}/\text{L}}}]}}{{v_{{{\text{coll}} t_{n} }}\, [{\text{L}}]}}$$

with

$$m_{{{\text{cp-stat}} t_{n} }} = m_{{{\text{P2NA}} t_{0} }} + \left( {m_{{{\text{P2NA }}t_{1} }} + \frac{1}{15}m_{{{\text{P2NA}} t_{0} }} } \right) + \cdots + \left( {m_{{{\text{P2NA}} t_{n} }} + \frac{1}{15}m_{{{\text{P2NA}} t_{0} }} + \cdots + \frac{1}{15}m_{{{\text{P2NA}} t_{n - 1} }} } \right)\;({\text{static}})$$

$$m_{{{\text{cp-dyn }}t_{n} }} = m_{{{\text{P2NA}} t_{0} }} + m_{{{\text{P2NA}} t_{1} }} + \cdots + m_{{{\text{P2NA}} t_{n} }} \;({\text{dynamic}})$$

and is presented in µg/cm^2^ to guarantee that results obtained in different laboratories can be directly compared with each other. Collected samples of static experiments were immediately replaced with equal amounts of receptor solution. All penetrated levels of P2NA at each subsequent time point (*t*_*n*_) were recalculated to correctly assess the total penetrated amount of P2NA according to the ratio between the collected volume (*v*_coll_, 0.5 mL) and the total volume (*v*_tot_, 7.5 mL) (=1/15). The % recovery of P2NA in the receptor fluid (Rec), the maximum flux (*f*_max_, µg/cm^2^/h), and the lag time (*t*_lag_, h) have also been determined (Harrison and Knutson 1995; Kupczewska-Dobecka et al. 2010).

## Results and discussion

The use of Franz diffusion cells is a well-established ex vivo model to study drug delivery in pharmacology and extensive information is available on pharmaceuticals (for review: Bartosova and Bajgar 2012; Godin and Touitou 2007). Similarly, Franz cells have been used in toxicology to study the transdermal absorption of organophosphates (Thors et al. 2016), glycol ethers (Larese Filon et al. 1999), polybrominated flame retardants (Frederiksen et al. 2016), and primary aromatic (di)amines such as aniline, *o*-toluidine, or 4,4′-methylenedianline (Lüersen et al. 2006; Kenyon et al. 2004). Less toxicological data are available on lipophilic compounds such as benzene (Adami et al. 2006) and polycyclic aromatic hydrocarbons (Bartsch et al. 2016), possibly due to their lower solubility in water and less pronounced skin penetration characteristics when compared to hydrophilic compounds.

Here, we show that P2NA, a highly lipophilic substance and secondary aromatic amine, can penetrate the skin although a previous study failed to show its transdermal absorption (Wellner et al. 2008). More importantly, P2NA accumulated in the subcutaneous layers of the skin from where it is continuously released to the receptor fluid of the Franz cells. This leakage of P2NA, in combination with its known in vivo conversion to 2NA, a potent bladder carcinogen in humans, can ultimately lead to toxicologically significant amounts of percutaneously absorbed P2NA.

### Successful standardization and pre-validation using primary aromatic amines

Both static and dynamic Franz cells were successfully standardized along the current OECD guideline 428 (OECD 2004). A constant physiological skin temperature (32 ± 1 °C) in all six Franz cells could be achieved using a thermostat (37 °C) for the circulating water bath and placing the porcine skin into the Franz cell 60 min prior start to achieve optimum equilibration. In case of dynamic experiments, the intra-cell (7.55 ± 0.05 mL/h, SD 2.6%) and inter-cell flow rates (7.55 ± 0.21 mL/h, SD 2.7%) was shown to be highly stable (*n* = 6).

The skin preparation using a dermatome was also reproducible (depth 956.87 ± 31.97 mm, *n* = 10) and the *SC* remained intact. The measured TEWL (*n* = 9) was similar between freshly prepared porcine ear skin (3.91 ± 0.67 g/cm^2^/h, SD 17.2%) and human skin of the inner and outer sides of the hands (4.43 ± 0.62 g/cm^2^/h, SD 14.0%), thus confirming the current OECD guidelines and previous studies showing that freshly prepared porcine ear skin can be used as a valid surrogate for human skin (OECD 2004; Bartek et al. 1972). No increase in TEWL could be observed up to 6 h after skin preparation (Tab. S1).

We pre-validated our system by measuring the transdermal absorption of less lipophilic primary aromatic amines, such as aniline and *o*-toluidine where data on skin penetration have been previously published (Wellner et al. 2008). However, instead of aniline, we used aniline-*d*_5_ to study its percutaneous absorption to avoid artefact contamination due to the omnipresence of aniline in our environment. All penetration parameters were normalized to an exposed area of 1 cm^2^, because the flux and the penetrated amounts of chemicals are dependent on the concentration and the exposed area (Michaels et al. 1975). Keeping in mind that we used freshly prepared porcine skin instead of frozen human skin, our data are well in line to those of Wellner and co-workers (Tab. S2).

### Optimizing Franz cell set-up minimizes losses of P2NA due to surface absorption

Non-polar analytes, such as P2NA are well absorbed on glass and plastic surfaces (Unger et al. 2001; Pekas 1972). Therefore, the experimental set-up was optimized to guarantee ideal study conditions. Special emphasis was placed on alternative receptor fluids along current OECD guidelines (OECD 2004), because a P2NA solution in 0.9% NaCl solution, previously used to study the ex vivo percutaneous absorption of P2NA (Wellner et al. 2008), resulted in considerable losses (recovery 30–69%) due to surface absorption on glass, PP, and PTFE, three commonly used materials in Franz cell experiments (Tab. S3a). However, surface absorption on glass was less pronounced than on PP and PTFE. Significantly increased recoveries (>90%, range 93–98%) were observed for EtOH/water (50/50, v/v) and 5% BSA in water (Tab. S3b). Consistently high recoveries of P2NA were also observed at different storage temperatures (21 vs. 37 °C), and upon testing multiple freeze/thaw cycles for both 5% BSA solutions (Tab. S3c/d) and EtOH/water (50/50, v/v) (data not shown). With the exception of scenario 3, we chose receptor solutions containing 5% BSA rather than 50% ethanol, based on the additional buffering capacity of BSA and its greater physiological relevance. In addition, high levels of EtOH might further enhance the dermal penetration of P2NA, and thus may result in misleading interpretations on the toxicological relevance of the percutaneous absorption of P2NA.

The right choice of gasket rings has also been identified as a critical parameter in our experiments. Teflon washers made out of Teflon foam rather than solid Teflon acted like a sponge and significantly absorbed P2NA from the donor solution (~30% of the applied dose). Consequently, fluororubber rings, an inert material, were used on the donor side to insert the porcine skin in Franz cells while further minimizing losses of P2NA.

### GC–MS/MS with isotope dilution allows accurate analysis of P2NA at low levels

Only few data are currently available on the analysis of P2NA. Limits of detection (LODs) in urine and physiological NaCl solution were reported to be high (~50 µg/L) due to the use of thin layer chromatography (Kummer and Tordoir 1975) or, despite the use of sensitive and specific GC–MS (Weiss and Angerer 2002), insufficient extraction, and derivatization of P2NA by diethylether and pentafluoropropionic anhydride (PFPA) (Wellner et al. 2008). In our studies, an excellent LOD (0.1 µg/L) and LOQ (0.3 µg/L) could be achieved by an efficient single liquid/liquid extraction of P2NA using *n*-hexane (extraction yield: 95%), the use of GC–MS/MS with splitless injection, and without any further derivatization of P2NA. The use of an isotope labelled ISTD (P2NA-d_5_) significantly improved the precision, accuracy, and robustness of the developed method. For example, the calibration curve was highly linear (2.5–2500 µg/L, *r* ^2^ = 0.996), thus covering a wide range of potentially penetrated concentrations of P2NA. No differences in the slopes of the calibration curves could be observed between 5% BSA and EtOH/water (50/50, v/v), which is in line with the results obtained during method development where nearly identical recoveries for P2NA were observed when both solutions were tested. Therefore, calibration standards were prepared in EtOH/water, whereas 5% BSA was used in the ex vivo experiments (Fig. S1). None of the reagent blanks spiked with P2NA-d_5_ contained traces of unlabelled P2NA. Consequently, the formation of artefacts from the use of the deuterium-labelled ISTD could be excluded.

The relative recovery (*n* = 10) of P2NA at 10 and 30 µg/L was 95–103% and 98–102%, respectively. The observed low intra-day and inter-day imprecisions (*n* = 9) at the same concentrations were 4.8–5.5% (10 µg/L) and 3.2–4.1% (30 µg/L), and are proof that this method delivers highly reproducible results in the ex vivo experiments and at low concentrations (Fig. S1b).

### Workplace-similar conditions show percutaneous absorption of P2NA at low levels

**Fig. 1:**
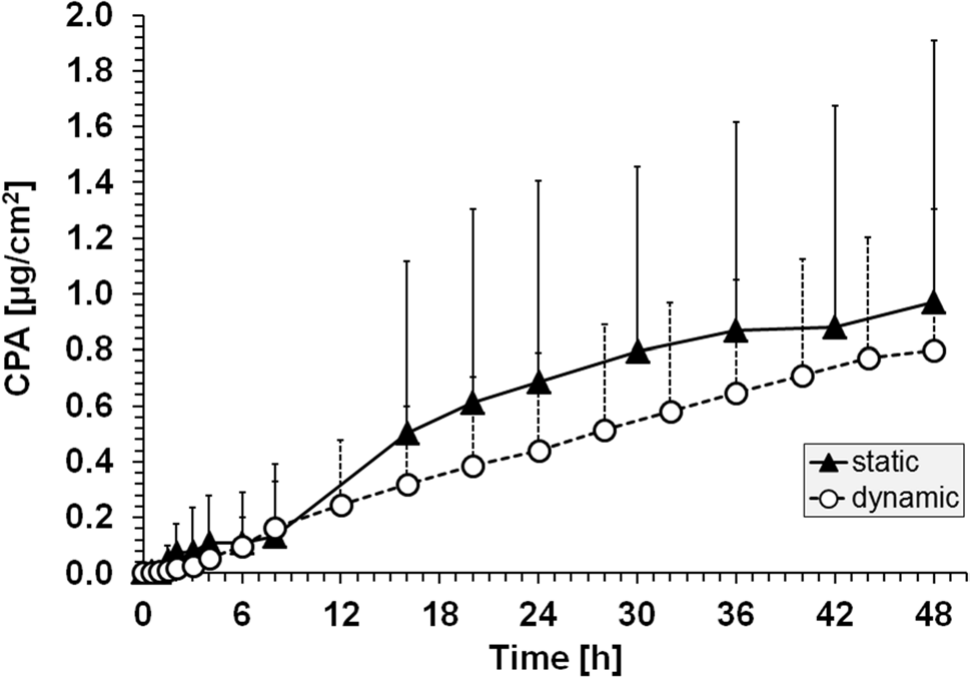
Cumulative penetrated amount (CPA) of P2NA using static and dynamic Franz cell conditions after application of P2NA (1 h, scenario 1); for the purpose of clarity, positive SD (*n* = 6) are included only

We were able to show that P2NA does penetrate the skin at very low levels when applying a donor solution of 12 g P2NA/L in 96% DCM and 4% corn oil for 1 h on the porcine skin (test scenario 1) (Fig. 1). For example, the CPA, the recovery of the applied dose, *f*_max_, and *t*_lag_ when using freshly prepared skin and dynamic conditions were 0.80 ± 0.26 µg/cm^2^, 0.04 ± 0.01%, 0.02 ± 0.01 µ/cm^2^/h, and 6.3 ± 2.2 h, respectively. Results using static conditions revealed similar penetration parameters (Table 1) showing that, in case of lipophilic chemicals such as P2NA, short application times and slow penetration characteristics allow the use of simpler static conditions to study percutaneous absorption, although saturation effects in the receptor fluid cannot be ruled out completely. Increased lag times and low recoveries of P2NA in the receptor fluid require, as already outlined in the OECD guideline (OECD 2004), an extended observation time of at least 48 h and an optimized receptor fluid to detect percutaneously absorbed amounts of lipophilic chemicals such as P2NA. When comparing aniline and P2NA at similar concentrations (aniline: 3 g/L, Tab. S1; P2NA: 12 g/L, this study, the same order of magnitude), the observed flux is about 1000 times lower for P2NA. Overall, our results show that the percutaneous absorption of P2NA can be efficiently studied in Franz cells using freshly prepared porcine skin.

**Table 1:** Mean and standard deviations of major skin penetration parameters of P2NA (scenario 1, 1 h application of P2NA, *n* = 6)

Franz cell	CPA (µg/cm^2^)	Rec (%)	*f*_max_ (µg/cm^2^/h)	*t*_lag_ (h)
Static	0.97 ± 0.75	0.05 ± 0.04	0.03 ± 0.02	7.90 ± 4.09
Dynamic	0.80 ± 0.26	0.04 ± 0.01	0.02 ± 0.01	6.33 ± 2.21

*CPA* cumulative penetrated amount, *Rec* recovery, *f*_*max*_ maximum flux, *t*_*lag*_ lag time

### Percutaneous absorption of P2NA is confirmed when following OECD guidelines

**Fig. 2:**
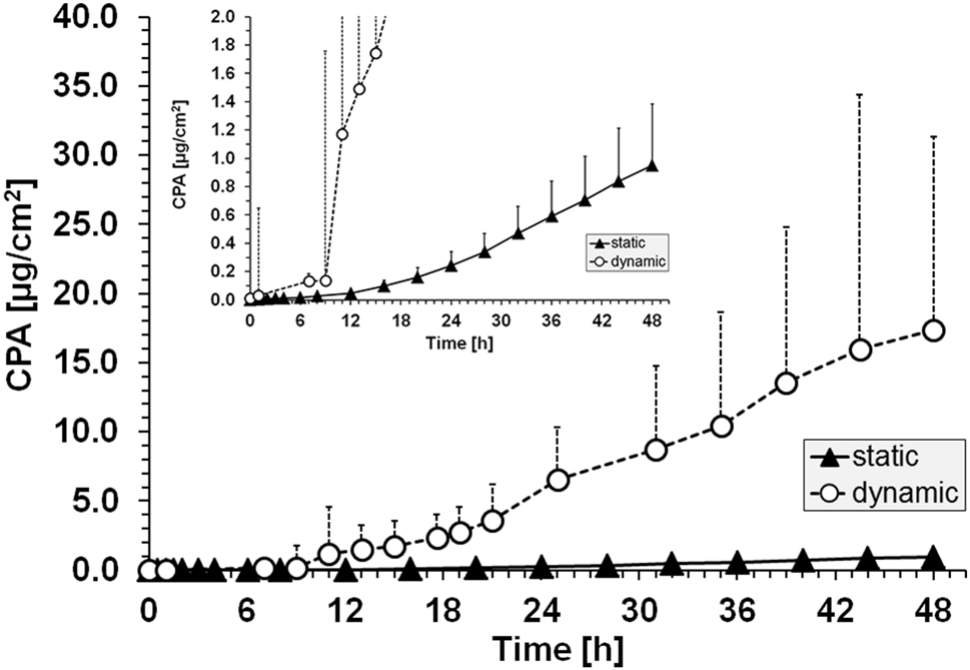
Cumulative penetrated amount (CPA) of P2NA using static and dynamic Franz cell conditions after application of P2NA (48 h, scenario 2); the inlet shows the identical diagram with a different *y*-axis intercept to make the SD for the static penetration curve visible; for the purpose of clarity, positive SD (*n* = 6) are included only

When we extended the application time to 48 h in compliance with OECD guidelines (scenario 2), we confirmed the percutaneous absorption of P2NA (Fig. 2; Table 2). Dynamic conditions revealed higher penetration parameters compared to static conditions (CPA: 17.40 ± 2.09 vs. 0.95 ± 0.43; *f*_max_: 0.55 ± 0.42 vs. 0.03 ± 0.01; *t*_lag_: 12.23 ± 1.09 vs. 11.64 ± 5.02; Rec: 0.91 ± 0.43 vs. 0.05 ± 0.02), most likely based on a constant exchange of the receptor fluid beneath the skin. Therefore, no rate-limiting effects, such as saturation of P2NA in the receptor fluid can occur (Bronaugh and Stewart 1985). Similar differences were observed when comparing the guideline-specific results to those of the workplace-similar results under dynamic conditions. Higher penetration parameters in the guideline-specific results can be explained by the longer application time of the substance (48 vs. 1 h). Interestingly, this difference could not be observed under static conditions, possibly due to saturation effects at the skin/receptor fluid interface and despite magnetic stirring (Díez-Sales et al. 1991). There, nearly identical results were observed for all penetration parameters between guideline-specific and workplace-similar results (Tables 1, 2) with the exception of *t*_lag_ which was slightly higher when following the OECD guideline (*t*_lag_ 11.6 vs. 7.9 h). This difference is most likely a mathematical artefact based on the fact that *t*_lag_ is calculated by extrapolating the slope of the penetration curve at *f*_max_ rather than presenting a true difference of *t*_lag_ between both experiments.

**Table 2:** Mean and standard deviations of major skin penetration parameters of P2NA (scenario 2, 48 h application, *n* = 6)

Franz cell	CPA (µg/cm^2^)	Rec (%)	*f*_max_ (µg/cm^2^/h)	*t*_lag_ (h)
Static	0.95 ± 0.43	0.05 ± 0.02	0.03 ± 0.01	7.79 ± 1.58
Dynamic	17.40 ± 2.09	0.91 ± 0.42	0.55 ± 0.42	12.23 ± 1.09

*CPA* cumulative penetrated amount, *Rec* recovery, *f*_*max*_ maximum flux, *t*_*lag*_ lag time

The results of both scenarios 1 and 2 show that more reliable results can be achieved using dynamic conditions, possibly based on the improved agreement with physiological conditions (i.e., simulating P2NA removal via blood flow) and its greater stability and robustness. In particular, saturation effects can be prevented by using dynamic conditions, which in turn, makes dynamic conditions the preferred option to study the percutaneous absorption of lipophilic chemicals, specifically at increased application times (≥48 h).

### Percutaneous absorption is enhanced by dichloromethane

**Fig. 3:**
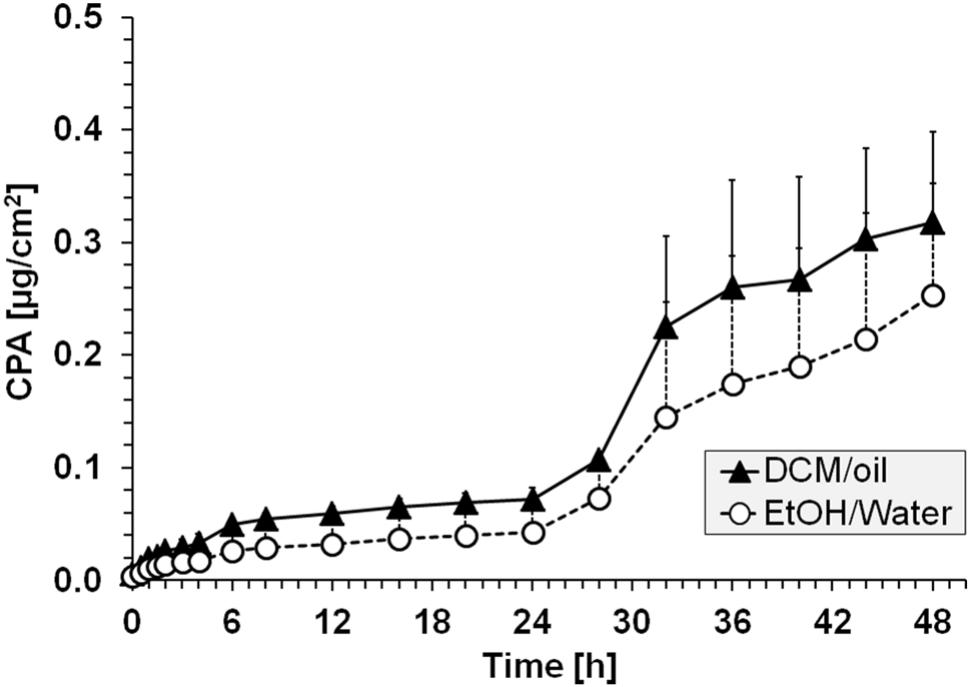
Cumulative penetrated amount (CPA) of P2NA in intact skin using dynamic Franz cell conditions after application of a 5 mg/L solution of P2NA (48 h, scenario 3) in 0.9% NaCl/5% ethanol/water and 96% DCM/4% corn oil; for the purpose of clarity, positive SD (*n* = 6) are included only

To directly study the influence of DCM/corn oil on the penetration of P2NA (scenario 3), it was necessary to use a lower concentrated and more aqueous solution of P2NA (5 mg/L, 0.9% NaCl +5% EtOH as solubilizer) as a reference. The results were directly compared to a 5 mg/L solution of P2NA in 96% DCM and 4% corn oil. Based on the aforementioned results, dynamic conditions and an application time of 48 h were chosen. A slightly increased percutaneous absorption was observed in the presence of DCM/corn oil, suggesting that DCM acts as penetration enhancer and facilitates the percutaneous absorption of P2NA (Fig. 3). However, an approximately 1.5-fold increase in CPA was observed after 48 h only (0.25 to 0.32 µg/cm^2^) (Table 3), whereas the penetration-enhancing properties must be considered negligible at shorter observation times. The solvent enhancing effect was confirmed based on slightly higher recovery rates and a shortening in *t*_lag_. Nevertheless, *f*_max_ remained unchanged. The observed minor DCM-enhancing effects are possibly due to a “residual solvent effect” (5% EtOH as solubilizer in the reference solution), which decreases the differences between the P2NA penetration curves in DCM/oil and 0.9% NaCl in EtOH/water. However, due to the poor solubility of P2NA in 100% water, no donor solutions without EtOH (true negative control) could be tested.

**Table 3:** Mean and standard deviations of major skin penetration parameters after application of P2NA (5 mg/L) in 0.9% NaCl/5% EtOH and 96% DCM/4% corn oil, an application time of 48 h, and under dynamic conditions (scenario 3, *n* = 6)

Donor	CPA (µg/cm^2^)	Rec (%)	*f*_max_ (µg/cm^2^/h)	*t*_lag_ (h)
0.9% NaCl/5% EtOH	0.25 ± 0.10	31.87 ± 12.57	0.01 ± 0.01	21.10 ± 1.83
96% DCM/4% corn oil	0.32 ± 0.08	56.23 ± 35.19	0.02 ± 0.01	16.77 ± 7.13

*CPA* cumulative penetrated amount, *Rec* recovery, *f*_*max*_ maximum flux, *t*_*lag*_ lag time

### P2NA accumulates in subcutaneous layers of the skin

International guidelines (COLIPA 1997; EPA 1999; SCCNEFP 1999; OECD 2004) with the exception of ECETOC (1993) all recommend the analysis and interpretation of the absorbed amounts in different cutaneous layers including the *SC*, epidermis, and dermis when studying the percutaneous absorption of lipophilic chemicals. However, only OECD (2004) and EPA (1999) recommend to include the *SC* in the interpretation of the results, whereas COLIPA (1997) and SCCNEFP (1999) do not, because they both consider chemicals in the *SC* as not bioavailable. Consequently, we analysed the *SC*, epidermis, and dermis of all skin samples at the time point of 48 h.

A direct comparison of the amounts of P2NA in the receptor fluids to those in the skin indicated an approximately 10–40-fold higher levels in the skin at 48 h (Tab. S4). Despite varying results, most of the percutaneously absorbed P2NA is observed in the dermis, followed by the *SC* and the epidermal fraction (Table 4). As expected, higher %-recoveries of P2NA were observed in the skin at 48 h compared to 1 h application times, and under both static (5.11 ± 3.53 vs. 0.93 ± 0.45) and dynamic conditions (5.53 ± 2.06 vs. 3.51 ± 1.78). Similarly, significantly increased penetrated amounts were observed in the skin samples derived from the experiments at higher concentrations (at 12 g/L: ~105 µg P2NA/cm^2^ when adding up the amounts of P2NA in *SC*, epidermis, and dermis) compared to lower concentrations (at 5 mg/L: ~13 µg/cm^2^). Conversely, the %-recoveries were significantly decreased at higher concentrations (12 g/L: 5.53 ± 2.06; 5 mg/L: 16.08 ± 13.75), because P2NA is less prone to reside in the skin. The results suggest a non-linear relationship between the applied concentrations and the penetration parameters for lipophilic substances, whereas a linear relationship has been observed when using less lipophilic primary aromatic amines such as aniline (Wellner et al. 2008).

**Table 4:** P2NA in various cutaneous layers of intact skin in terms of the penetrated amounts (µg/cm^2^) and the recovery (%) at the end of all experiments (time point 48 h)

Franz cell	PA_sc_ (µg/cm^2^)	PA_epi_ (µg/cm^2^)	PA_derm_ (µg/cm^2^)	Rec (%)
Scenario 1: 12 g/L P2NA^a^; application 1 h				
Static	9.52 ± 5.74	1.02 ± 0.35	7.20 ± 2.53	0.93 ± 0.45
Dynamic	7.37 ± 5.22	24.92 ± 13.56	34.68 ± 15.19	3.51 ± 1.78
Scenario 2: 12 g/L P2NA^a^; application 48 h				
Static	38.18 ± 35.29	12.23 ± 8.21	47.23 ± 23.86	5.11 ± 3.53
Dynamic	32.60 ± 12.53	29.62 ± 14.82	43.46 ± 12.02	5.53 ± 2.06
Scenario 3: 5 mg/L P2NA^a,b^; application 48 h				
Dynamic^a^	1.40 ± 1.10	3.66 ± 4.23	7.81 ± 4.04	16.08 ± 13.75
Dynamic^b^	1.33 ± 0.77	0.82 ± 0.56	5.70 ± 4.04	9.81 ± 11.72

*PA*_*sc*_ *, PA*_*epi*_ *, PA*_*derm*_ penetrated amount in *stratum corneum*, epidermis, and dermis, *Rec* recovery

^a^In 96% DCM/4% oil

^b^In 0.9% NaCl/5% EtOH

At identical concentrations (5 mg/L), higher amounts of P2NA were observed in the skin and the receptor fluid when using a solution of 96% DCM/4% oil compared to 0.9% NaCl in 5% EtOH/water (Tables 3, 4). The same is true for the %-recoveries. For example, the %-recovery in skin (9.8%) and the receptor fluid (31.9%) is lower in case of the 5 mg/L solution of P2NA in 0.9% NaCl/5% EtOH compared to the solution in 96% DCM/4% oil (16.1 and 56.2%, respectively). These results, despite similar *f*_max_ in both experiments (~0.01–0.02 µg/cm^2^/h), further demonstrate a penetration-enhancing effect of DCM. This effect is most likely based on the high skin permeability of DCM itself rather than its skin-damaging potential. Although an irritating effect of DCM on the skin (in particular on *SC*) cannot be completely ruled out, our results show that such a damage is unlikely due to a similar shape of the penetration curves with and without DCM (Fig. 3). In addition, the amount of P2NA in the *SC* was similar with and without DCM (Table 4). Consequently, we assume that DCM “pushes P2NA through the intact skin” based on its own high skin permeability and does not allow P2NA to interact with cutaneous structures, an assumption which is further supported by the observed decreased *t*_lag_ in DCM/oil compared to EtOH/water.

### The combination of percutaneous absorption and accumulation in skin can result in toxicologically significant amounts of P2NA

Our results clearly show that the most reliable results for the percutaneous penetration of P2NA are observed when a combination of dynamic Franz cells and freshly prepared porcine skin is used. The CPA, recovery, and *f*_max_ in scenario 1, which most closely resembles the former workplace situation in the 1960s/1970s in the printing industry (*c* = 12 g/L, *t* = 1 h) were 0.80 ± 0.26 µg/cm^2^, 0.04 ± 0.01%, and 0.02 ± 0.01 µg/cm^2^/h, respectively. Conversely, 1000-fold higher *f*_max_ values (>20 µg/cm^2^/h) could be found for primary aromatic amines, such as aniline and *o*-toluidine at similarly high concentrations (≥3 g/L, Wellner et al. 2008). Therefore, dermal exposure of P2NA appears to be very low, and at first glance, suggests an only minor contribution to the total internal body burden (and consequently bladder cancer risk) of workers, despite its conversion to highly carcinogenic 2NA in vivo.

**Fig. 4:**
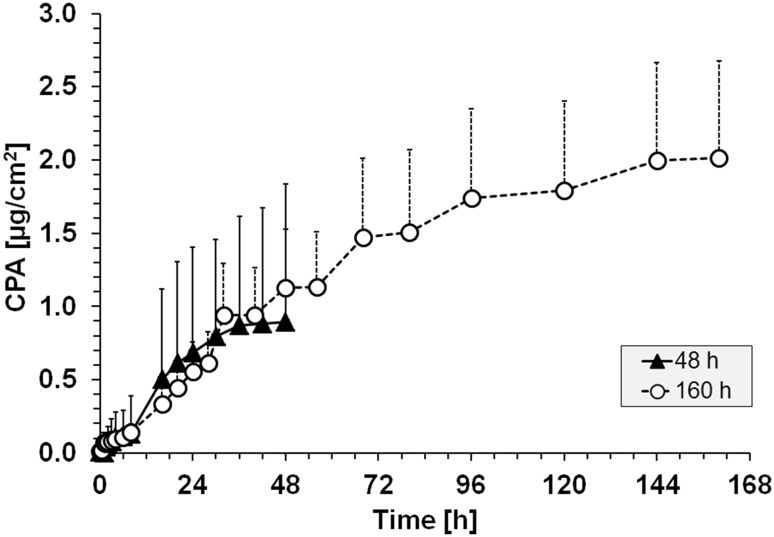
Percutaneous absorption of P2NA (12 g/L in 96% DCM/4% corn oil) up to 160 h under static conditions, an application time of 1 h, and compared to the results obtained in the initial experiment (scenario 1) with an observation time of 48 h only

However, the results of scenario 1 also show a constant increase in the cumulative penetrated amount of P2NA up to 48 h, although the donor solution was removed after 1 h (Fig. 1). In combination with the observed accumulation in skin, we assumed that P2NA is constantly released from the subcutaneous layers of the skin. Therefore, we repeated the experiment and extended the observation time up to 160 h. Again, the results show a clear and constant increase in the CPA of P2NA (Fig. 4) despite the removal of the P2NA-containing donor solution after 1 h. The results further strengthen the assumption that P2NA is continuously released from subcutaneous skin layers, and consequently further contributes to the internal body burden of P2NA in workers despite exposure cessation.

Overall, our results clearly show that close attention must be paid to dermal absorption of P2NA in risk assessment of formerly exposed workers and specific exposure circumstances, such as the presence or absence of penetration enhancers and the potential accumulation of a substance in the skin, which needs to be addressed. Furthermore, non-occlusive exposure circumstances including the rapid evaporation of DCM must be taken into account, which may additionally influence dermal uptake of P2NA at workplaces.

## Electronic supplementary material

Below is the link to the electronic supplementary material.

Supplementary material 1 (DOCX 67 kb)
