# Correction to: Quantification of *N*-phenyl-2-naphthylamine by gas chromatography and isotope-dilution mass spectrometry and its percutaneous absorption ex vivo under workplace conditions

**DOI:** 10.1007/s00204-017-2095-6

**Published:** 2017-10-31

**Authors:** Eike Maximilian Marek, Stephan Koslitz, Tobias Weiss, Manigé Fartasch, Gerhard Schlüter, Heiko Udo Käfferlein, Thomas Brüning

**Affiliations:** Institute for Prevention and Occupational Medicine of the German Social Accident Insurance, Ruhr-University Bochum (IPA), Burkle-de-la-Camp Platz 1, 44789 Bochum, Germany

## Correction to: Arch Toxicol DOI 10.1007/s00204-017-2046-2

The article ‘Quantification of *N*-phenyl-2-naphthylamine by gas chromatography and isotope-dilution mass spectrometry and its percutaneous absorption ex vivo under workplace conditions’ written by Heiko Udo Käfferlein, was originally published electronically on the publisher’s internet portal (currently SpringerLink) on 12th September 2017 without open access.

With the author(s)’ decision to opt for Open Choice the copyright of the article changed on 17th October 2017 to © The Author(s) 2017 and the article is forthwith distributed under the terms of the Creative Commons Attribution 4.0 International License (http://creativecommons.org/licenses/by/4.0/), which permits use, duplication, adaptation, distribution and reproduction in any medium or format, as long as you give appropriate credit to the original author(s) and the source, provide a link to the Creative Commons license and indicate if changes were made.

