# Supplementary material for: Quantification of N-phenyl-2-naphthylamine by gas chromatography and isotope-dilution mass spectrometry and its percutaneous absorption ex vivo under workplace conditions
Source: Arch Toxicol. 2017 Sep 12;91(11):3587–96. doi: 10.1007/s00204-017-2046-2 (PMC5696485; doi:10.1007/s00204-017-2046-2)
Supplement: Supplementary file 1 — Supplementary material 1 (DOCX 67 kb) [file 204_2017_2046_MOESM1_ESM.docx]

**SUPPLEMENTAL FIGURES AND TABLES**

**Fig. S1.** Chromatograms of **(a)** standard solutions (lift side) containing 2.5 (dashed), 10 (dotted) and 30 µg P2NA/L (continuous) in ethanol/water (50/50, v/v) and the corresponding internal standard (right side) and **(b)** receptor solutions (5% BSA) from the time points 1 h (dashed), 18h (dotted) and 48 h (continuous) of a Franz cell of scenario 2 (static, 48 h exposition).

**(a)** **(b)**

**Tab. S1.** Transepidermal water loss (TEWL) of intact porcine skin up to 48 h (n=9 skin surface areas); SD, standard deviation.

|  |  |  |
| --- | --- | --- |
| **Time** | **TEWL** | **SD** |
| Fresh (“0 h”) | 4.78±0.62 | 13.0% |
| 6 h | 4.67±0.65 | 14.0% |
| 24 h | 5.01±0.68 | 13.6% |
| 48 h | 5.12±0.64 | 12.5% |

**Tab. S2.** Cumulative penetrated amount (CPA), recovery (Rec), maximum flux (f_max_) and lag-time (t_lag_) of o-toluidine and aniline using comparable Franz cell conditions as previously published (Wellner at al. 2008), i.e. application and observation time 24 h, donor solutions of aromatic amines in 0.9% NaCl / 5% ethanol; receptor solution, 0.9% NaCl.

| **Test Substance** | **CPA [µg*cm^-2^]** | | **Rec [%]** | **f_max_ [µg*cm^-2^*h^-1^]** | **t_lag_ [h]** |
| --- | --- | --- | --- | --- | --- |
| **30.0 mg/L o-toluidine** | | | | | |
| Wellner *et al.*^a^ (*n*=9) | | 7.47±0.47 | 49.79±3.16 | 0.37±0.04 | 0.80±0.10 |
| This study^b^ (*n*=6) | | 2.22±0.11 | 46.58±2.36 | 0.25±0.04 | 0.37±0.14 |
| **pure *o*-toluidine** | | | | | |
| Wellner et al. (*n*=4) | | 415.16±110.37 | 0.08±0.02 | 117.04±28.33 | 2.50±0.30 |
| This study (*n*=6) | | 9.846.76±828.70 | 2.00±0.20 | 850.00±4.22 | 1.65±0.40 |
| **30.0 mg/L aniline or aniline-d_5_** | | | | | |
| Wellner *et al.*^c^ (*n*=8) | | 5.65±0.24 | 37.64±1.59 | 0.30±0.02 | 0.80±0.10 |
| This study^d^ (*n*=6) | | 4.56±0.81 | 95.54±17.03 | 0.50±0.06 | 0.23±0.16 |
| **3,000 mg/L anilin or aniline-d_5_** | | | | | |
| Wellner *et al.*^e^ (*n*=4) | | 466.60±64.11 | 28.91±4.27 | 18.40±2.34 | 1.10±0.20 |
| This study^f^ (*n*=6) | | 199.35±28.80 | 41.75±6.03 | 29.74±4.35 | 0.31±0.17 |
| Applied amount: ^a^150 ng/cm^2^, ^b^48 ng/cm^2^, ^c^150 ng/cm^2^, ^d^48 ng/cm^2^, ^e^15 µg/cm^2^, ^f^4,8 µg/cm^2^ | | | | | |

Tab. S3. Recovery of P2NA [%] after (a) dissolution in aqueous 0.9% NaCl at three different concentrations and storage in glass, polypropylene (PP) and polytetrafluoroethylene (PTFE) vessels; (b) in different receptor fluids at a concentration of 5 mg P2NA/l; (c) at different storage and treatment conditions using a 5 mg P2NA/l solution containing 5% bovine serum albumin (BSA) and up to 24 h, and (d) after freezing and four freeze/thaw cycles up to six days.

| **(a)** |  |  |  |  | **(b)** |  |  |  |
| --- | --- | --- | --- | --- | --- | --- | --- | --- |
| **Concentration** | **Glass** | **PP** | **PTFE** |  | **P2NA in...** | **Glass** | **PP** | **PTFE** |
| 10 µg/l | 59% | 44% | 49% |  | 0,9% NaCl | 52% | 30% | 39% |
| 200 µg/l | 69% | 41% | 44% |  | 5% BSA | 98% | 94% | 95% |
| 5,000 µg/l | 52% | 30% | 39% |  | 50% Ethanol | 94% | 95% | 93% |

| **(c)** |  |  |  |  |  |  |  |
| --- | --- | --- | --- | --- | --- | --- | --- |
| **Storage at...** | **Vessel** | **Stirring** | **0.5 h** | **1.0 h** | **2.0 h** | **4.0 h** | **24.0** |
| 37°C | Glass | Yes | 91% | 90% | 89% | 96% | 90% |
|  |  | No | 94% | 90% | 91% | 90% | 94% |
| 21°C (RT) | Glass | No  No | 83% | 84% | 84% | 83% | 84% |
|  | PP |  | 79% | 79% | 77% | 76% | 73% |

| **(d)** |  |  |  |  |  |
| --- | --- | --- | --- | --- | --- |
| **Vessel** | **Treatment** | **24 h** | **48 h** | **72 h** | **144 h** |
| Glass | Freeze | 104% | 95% | 91% | 94% |
|  | Freeze/thaw | 101% | 92% | 90% | 98% |
| PP | Freeze | 99%  95% | 101% | 93% | 87% |
|  | Freeze/thaw |  | 101% | 103% | 91% |

**Tab. S4.** %-Recoveries of P2NA in the receptor fluids (Rec_RF_) and directly compared to those obtained in intact skin (Rec_SK_, n=6).

| **Franz cell** | **Rec_RF_[%]** | **Rec_SK_[%]** |
| --- | --- | --- |
| Scenario 1: 12 g/L P2NA^1^; application 1 h | | |
| static | 0.05±0.04 | 0.93±0,45 |
| dynamic | 0.04±0.01 | 3.19±1,87 |
| Scenario 2: 12 g/L P2NA^1^; application 48 h | | |
| static | 1.49±1.18 | 5.53±2,06 |
| dynamic | 0.05±0.02 | 5.11±3,53 |
| Scenario 3: 5 mg/L P2NA^1,2^; application 48 h | | |
| dynamic^1^ | 31.87±12.57 | 16.08±13,75 |
| dynamic^2^ | 56.23±35.19 | 9.81±11,72 |
| ^1^ in 96% DCM / 4% oil, ^2^ in 0.9% NaCl/5% EtOH | | |
